# Surgical instrument tray optimization process at a university hospital: A comprehensive overview

**DOI:** 10.1016/j.sopen.2024.09.007

**Published:** 2024-10-05

**Authors:** Peter Rubak, Ann-Eva Christensen, Mads Granlie, Karin Bundgaard

**Affiliations:** a Clinical Nursing Research Unit, Aalborg University Hospital, Aalborg, Denmark; b Unit for Psychiatric Research, North Denmark Region, Aalborg, Denmark; c Acquisition and Logistics Organization, process and digitalization department, Denmark; d Department of Clinical Medicine, Aalborg University, Aalborg, Denmark

**Keywords:** Healthcare costs, Surgical instrument tray optimization, Resource utilization, Quality improvement

## Abstract

**Objective:**

This study presents the results of a surgical instrument tray optimization process implemented across all surgical specialties within the largest university hospital in Denmark.

**Methods:**

Data was extracted from a comprehensive instrument optimization process including all Operating Rooms at Aarhus University Hospital. Adopting a holistic perspective, the optimization process, involved aligning instrument trays across various surgical specialties. This included: a) Reduction in number of instruments, b) Consolidation or separation of trays, c) Modularization - introducing modular trays for specific purposes, and d) Standardization - standardizing commonly used instruments across specialties. Instruments per tray, total number of instruments, and changes in the number of trays were compared against existing tray contents to identify discipline-specific changes.

**Results:**

Some specialties made substantial alterations to tray structures, while others primarily reduced number of instruments in existing trays. Across all specialties, optimization resulted in 17 % decrease in number of tray types (*p* = 0.01, 95%CI:1.0–6.8), 1 % increase in total number of trays (*p* = 0.36, 95%CI:-11.9–4.8), 18 % decrease in number of instruments per tray (*p* = 0.0002, 95%CI: 3.2–7.6) and 16 % reduction in total number of instruments for all specialties (*p* < 0.0001, 95%CI:404–758).

**Conclusion:**

This study underscores complexity of instrument tray design. The approach employed at Aarhus University Hospital, involving interdisciplinary experts in an iterative design process, demonstrated the feasibility of redesigning instrument trays with significant reduction in content. Additionally, data suggests that reducing the number of instruments could lead to a decrease in workload within the Central Sterile Supply Specialty. This presents opportunity to minimize wasted resources and streamlining cleaning processes for unused instruments.

## Highlights

• Tray optimization is complex.
• It is feasible to redesign instrument trays with significant reduction in content.
• A structured iterative design involving experts supports the optimization process.
• Total number of instruments is more accurate than using instruments per tray.
• Tray optimization may minimize wasted resources and streamline cleaning processes.

## Introduction

Healthcare costs are on the rise both nationally and internationally, with Denmark experiencing a 69 % increase in healthcare costs from 2000 to 2017 [1].

One of the primary drivers of healthcare expenditure globally is the hospital setting, particularly the resource utilization in operating rooms (ORs) [2]. Studies indicate that up to 56 % of perioperative costs are associated with material and supply utilization, including both reusable and single-use items [3,4]. Even minor changes in efficiency of operating room processes can significantly impact healthcare spending, emphasizing the need to scrutinize work processes and resource utilization in this setting.

In recent years, surgical instrument tray optimization has emerged as a method to reduce perioperative costs [5]. This reduction is achieved by minimizing setup time in the OR [6], processing time [7], and tray preparation time [8]. Optimizing instrument trays has the potential to cut costs, enhance efficiency, increase capacity, and support quality improvements.

Over time, instrument trays have evolved to align with surgeons' preferences and adjust to various surgical specialties and specific procedures. The focus has predominantly been on adding new instruments to adapt advancements in surgical methods. However, there has been limited attention to removing instruments that are no longer necessary resulting in trays with unused or rarely used instruments [9]. For instance, advancements in electrocautery techniques have reduced the need for traditional haemostatic instruments [10]. Studies in spine and gynecology surgery reveal usage rates ranging from 20.5 % to 58 %, indicating a significant portion of instruments in trays being underutilized. Reprocessing unused instruments encounters additional expenses of approx. 3.19 USD for each unused instrument and contributes to resource wastage, as labor, energy, and time are invested in the process [4,11]. Furthermore, the repeated washing and sterilization processes can lead to wear and tear, necessitating the replacement of instruments that have not been frequently used [12].

Existing literature on optimization processes predominantly focuses on numbers and types of instruments in the trays, where specialty-specific surgeons decide on the removal of instruments tray by tray. A scoping review by Dos Santos et al. [13] reported reductions >50 % in 20 studies, reductions between 26 and 50 % in nine studies and reductions below 25 % in seven studies, emphasizing the specialty-specific nature of these processes. Farrelly et al.'s single site study, reported an average elimination of 34.0 % of instruments per tray, with complete removal of nine out of 21 trays and a reduced tray weight [5]. Another study demonstrated a 41 % reduction in instruments across three general thoracic surgery departments [14].

However, existing literature predominantly addresses optimization within single or a few departments, leaving uncertainty about the applicability of the same process across multiple surgical departments or an entire hospital. Therefore, this paper aims to present the outcomes of a surgical instrument tray optimization implemented across all surgical specialties at the largest University Hospital in Denmark.

## Material and methods

### Study design

This study employs a case study approach, utilizing statistical analysis and data obtained from an instrument optimization process encompassing all operating rooms and production data from the Central Sterile Supply Department (CSSD) at a University Hospital.

### Setting

The optimization process took place at Aarhus University Hospital, the largest university hospital in Denmark, employing approximately 10.000 staff members. The hospital has 74 operating rooms distributed across five departments with the specialties Neuro and Spine (NKX), Orthopedics (EOP), Otolaryngology (HOP), Odonto-maxillary (TMK), Pediatrics (BUO), Obstetrics and Gynecology (YOP), Plastic (ZOP), Cardio-pulmonary and Vascular (TOP), Gastroenterology (MTK), Urology (KOP), and Same Day Surgery (DKA) and Ophthalmology. Annually, the hospital performs approximately 60.000 surgeries, excluding those conducted in the Ophthalmology specialty, which operates within its own sterile processing and instrument circulation system. The hospital's CSSD is responsible for the reprocessing of reusable surgical instruments, as well as the packaging and distribution of single-use items, procedure packs, instrument trays, and peel pack instruments for surgery. The CSSD is staffed by 150 persons, including 55 warehouse staff, 80 reprocessing staff, 10 nursing specialists, and 10 other staff members, including management.

In 2019, Aarhus University Hospital embarked on a comprehensive surgical instrument tray optimization process including all surgical disciplines. A thorough inventory analysis of existing instruments, along with an assessment of reprocessing capacity, revealed a multitude of worn-out instruments and highlighted the necessity for additional capacity in reprocessing and tray numbers. This analysis showed that 34 % of the scissors and scalpels category were functional, 18 % required repair or surface treatment, and 48 % needed to be replaced. Similar numbers were seen for forceps with 27 % were functional, 43 % required repair or surface treatment, and 30 % needed to be replaced. Significant differences were observed across specialties. TOP had the most worn-out instruments, with only 28 % being functional, 30 % requiring repair, and 45 % needing replacement, compared to the NKX, which had the highest quality instruments, with 74 % functional, 15 % needing repair, and 11 % requiring replacement. A total of 92 different manufacturers of open surgery instruments were identified in the sample, with the 5th largest manufacturer categorized as “unknown” due to wear, reflecting the age of the instruments by lacking instrument ID or other identification markings. To focus scope, reduce complexity and costs, only instruments for open surgery were selected for optimization. In total, 1340 different instrument trays were selected for optimization.

### Design approach

The design of the new instrument trays was conducted through a collaborative workshop involving one surgeon and one OR nursing specialist from each surgical discipline all of whom had extensive experience within the specialty. Additionally, one consultant from the instrument manufacture with extensive knowledge of instruments and two facilitators from the hospital quality department provided support, employing a structured approach comprised of five interconnected elements, as delineated below. These elements were integrated into an iterative design process aiming to reduce the number of instruments by redesigning the tray structure. During this process, focus was on reaching consensus instead of individual preferences. To facilitate this process, a diverse array of instruments was made available for thorough review.

#### Holistic view

All existing tray types from each specialty were collectively reviewed during a workshop session to establish an understanding of overlaps, similarities, differences, and potential combinations of instrument trays.

#### Reduction

Instruments that were either unused or utilized less frequently than once in every fourth or fifth operation were systematically removed from the trays. This decision was solely based on the workshop surgeons and OR nurses' assessment. Exceptions were made for instruments required in acute or unpredictable scenarios (e.g., vascular clamps), which were retained or converted to peel pack format.

#### Consolidation or separation

Trays with identical contents, except for a few instruments, were consolidated based on consensus among clinical specialists. Conversely, separation was applicable when a group of instruments served specific and predictable purposes. For example, trays for shoulder and elbow surgery were segregated into three distinct trays: one for shared instruments, one for shoulder-specific instruments, and another for elbow-specific instruments.

#### Modularization

Instruments specific to certain types of surgery were modularized, allowing for the creation of specialized modules within basic instrument trays. For instance, in plastic surgery, modules for post-bariatric surgery and breast reconstruction were designed.

#### Standardization

A selection of commonly used instruments, both within and across specialties, was designated as the ‘gold standard’. This included instruments such as tissue clamps, needle holders, and forceps. However, accommodations were made for specialized instruments as deemed necessary.

### Data collection

Data on the newly designed instrument trays was gathered during the workshops. The following metrics were collected and compared against the existing trays and their contents for each individual specialties to determine reductions per discipline:

• Tray Types**:** Total number of unique types of trays
• Total Number of Trays**:** Total number of trays across all specialties
• Instruments per Tray Type**:** Number of instruments in each tray type
• Total Number of Instruments**:** Total number of instruments present in all trays

### Statistics

Number of tray types, total number of trays, instruments per tray type as well as total number of instruments are presented as before and after optimization for each specialty, and the relative reduction is calculated. To test for a general tendency across specialties, a *t*-test on the reduction in number of tray types, total number of trays, instruments per tray type and total number of instruments were conducted. The results are considered to be statistically significant if *P* < 0.05.

## Results

### Redesign

The redesign of instrument trays led to substantial reductions in the number of required instruments. Several specialties within the hospital made extensive changes to their tray structures. For instance, YOP and EOP reduced their number of tray types by 33 % and 25 %, respectively, while others, such as BUO, primarily focused on reducing the number of instruments within their existing trays. Overall, across all specialties, the number of tray types decreased from 258 before optimization to 215 after optimization, representing a reduction of approximately 17 % (Range: 0–33 %). Which corresponds to a statistically significant average reduction of 3.9 tray types per specialty (*p* = 0.01, 95 % CI: 1.0–6.8), Table 1.

**Table 1:** Results before and after instrument tray optimization shown per specialty.

Specialty	Before optimization				After optimization				Results			
	Tray types	Total no. of trays	Instruments per tray^a^	Total no. of instruments	Tray types	Total no. of trays	Instruments per tray^a^	Total no. of instruments	Tray types	Total no. of trays	Instruments per tray	Total no. of instruments
BUO	15	82 [1−21]	32,0	2.338	15	83 [1–21]	25,5	2.080	0 %	1 %	-20 %	−11 %
DKA	38	217 [1−22]	28,0	6.320	35	224 [1–22]	25,3	5.492	−8 %	3 %	−10 %	−13 %
EOP	64	269 [1–18]	29,5	8.164	48	278 [1−30]	25,4	7.449	−25 %	3 %	−14 %	−9 %
HOP	26	135 [1–26]	26,8	4.759	22	135 [1−20]	22,2	3.943	−15 %	0 %	−17 %	−17 %
KOP	16	73 [1−10]	30,8	2.589	16	83 [1−12]	20,2	2.195	0 %	14 %	−34 %	−15 %
MTK	18	107 [1–16]	30,6	3.850	15	100 [1–24]	22,1	2.914	−17 %	−7 %	−28 %	−24 %
NKX	18	85 [1–16]	23,7	2.723	14	88 [1–16]	21,9	2.570	−22 %	4 %	−8 %	−6 %
TMK	11	55 [1–20]	33,6	1.952	9	48 [1–20]	30,3	1.507	−18 %	−13 %	−10 %	−23 %
TOP	18	112 [1–20]	44,9	5.040	16	110 [1–20]	38,1	4.436	−11 %	−2 %	−15 %	−12 %
YOP	12	56 [1−13]	26,1	1.621	8	46 [2−10]	25,3	1.214	−33 %	−18 %	−3 %	−25 %
ZOP	22	127 [1–45]	22,5	3.717	17	162 [2–40]	12,9	2.887	−23 %	28 %	−42 %	−22 %
Total	258	1.318	328,4	43.073	215	1.357	269,1	36.687	−17 %	1 %	−18 %	−16 %

a Mean number of instruments per tray, [] = range of number of unique tray types.

**Fig. 1:**
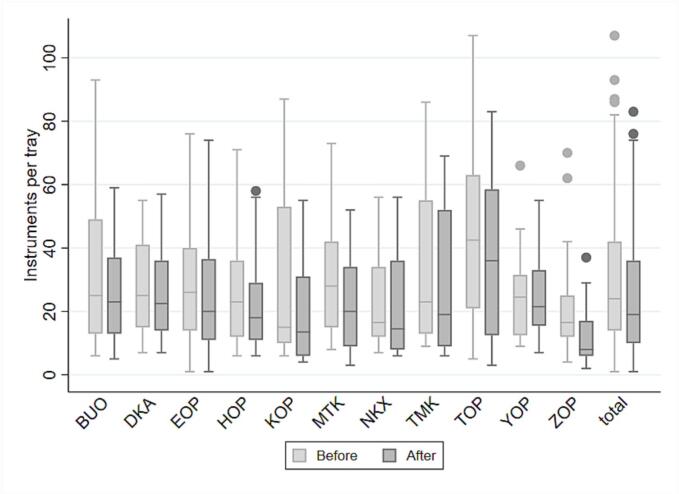
Distribution of instruments per tray before and after optimization visualized for each specialty. This figure illustrates the reduction in the number of instruments per unique tray type before and after the optimization process, both for each specialty and in total.

Furthermore, the optimization efforts resulted in a reduction in the number of instruments per tray across all specialties from a mean number of 30 before optimization to 24 after optimization, indicating an approximate 18 % reduction (Range: 3–42 %). Which corresponds to a statistically significant average reduction of 5.4 instruments per tray per specialty (*p* = 0.0002, 95 % CI: 3.2–7.6), Fig. 1.

While the total number of trays increased from 1318 before optimization to 1357 after the optimization process, corresponding to approximately 1 % (Range: −18-28 %), which corresponds to an average increase of 3.5 total number of trays per specialty. However, the result is not statistically significant (*p* = 0.36, 95 % CI: −11.9–4.8). There was a decrease in the total number of instruments across all specialties from 43.073 instruments before optimization to 36.687 after optimization, reflecting a reduction of approximately 16 % (Range: 6–25 %). Which corresponds to a statistically significant average reduction of 581 instruments per specialty (*p* < 0.0001, 95 % CI: 404–758), Table 1.

All trays registered as having 0 number of instruments pr. tray has been eliminated in both Table 1 and Fig. 1.

### Holistic view

Taking a holistic view proved instrumental in achieving comprehensive optimization across all disciplines. The most significant transformation occurred in EOP, where trauma and elective hip/knee surgery trays were aligned. Purpose-specific trauma trays were eliminated, replaced by two sets of trays designed to basic surgical needs: one for large bone surgeries and another for small bone surgeries. Specialized trays for pediatric, reconstructive, and oncological surgeries were redesigned to align with this new tray structure, eliminating redundancies. Overall, these changes in EOP resulted in a reduction of tray types from 64 to 48 (25 %). Although the total number of trays increased from 269 to 278 (approx. 3 %), there was a decrease in the total number of instruments from 8164 to 7449 (approx. 9 %).

### Reduction

Across all specialties, there was a general reduction in the number of instruments per tray, leading to a reduced tray complexity. Fig. 1. Four specialties - TOP, TMK, YOP, and HOP - primarily kept their existing tray structures. However, more than half of the specialties maintained their existing number of trays while reducing the number of instruments by 6 % to 24 %. Conversely, KOP and ZOP surgery reduced the number of instruments but increased their tray capacity by 14 % and 28 % respectively. This reduction in instruments was the result of redesigning and modularizing tray contents and purposes within these specialties.

### Consolidation or separation

Consolidation or separation was seen across all specialties. For example, consolidated MTK their lower and upper intestinal surgery basic trays into a single unit, while introducing supplementary trays specific to upper and lower intestinal surgeries. Previously, the “basic tray lower” was utilized for robotic surgery, although only 40 % of the instruments were needed if the surgery had to be converted into open surgery. Post-optimization, the newly merged basic tray is employed for robotic surgeries from the outset, with the “supplementary tray lower” accessible but opened only as needed. Overall, a total number of 59 different tray types were eliminated and 15 new tray types were introduced.

### Modularization

An example of modularization was seen in ZOP where they reduced their basic tray and introduced six modular trays for specific purposes, such as bariatric reduction and breast reconstruction, rather than incorporating all functionalities within the basic tray. Biopsy trays were subdivided into small and large trays, as the larger set of instruments was only required in certain situations.

### Standardization

Before the optimization, there was significant variation in sizes of commonly used instruments due to purchases made over several years from different manufacturers. Each specialty managed its own instrument inventory, further contributing to this variation, as preferences of individual surgeons were also accommodated. Through the optimization workshop, commonly used instruments were standardized hospital wide. For instance, the variety of forceps was reduced from 43 to 26 types post-optimization.

## Discussion

This study highlights the outcomes of the optimization process conducted at a University Hospital, demonstrating a reduction in total number of instruments and number of tray types across all specialties. However, there was a small increase in the total number of trays post-optimization.

Instrument tray design is complex, and there is no single approach guaranteed to minimize instrument usage. A scoping review by Dos Santos et al. [13] outlines various methods employed to reduce the number of instruments, ranging from expert analysis and Lean practices to mathematical programs and key-driver programs, as well as observations and value stream mapping. The approach adopted at Aarhus University Hospital integrated multiple methods, showcasing how interdisciplinary teams of experts engaged in an iterative design process successfully redesigned instrument trays, resulting in a substantial reduction in the total number of instruments.

Our study found a statistically significant average reduction of 18 % in instruments per tray across the hospital, with variations ranging from 3 % in YOP to 42 % in ZOP. These figures are lower compared to existing research. For example, Farrelly et al. [5] reported an average elimination of 34 % of instruments per tray in their single-site study at a pediatric surgical specialty, while Dekonenko et al. [7] removed 40 %–70 % of surgical instruments per tray in a similar setting. In this study the number of unique tray types were reduced in all surgical specialties except for KOP and BUO where the number of tray types was the same after optimization. However, it's crucial to be aware that our optimization process included consolidation, separation, modularization, and standardization, leading to changes in both composition and number of instruments within tray types. One explanation of the observed reduction in total instrument could be an increased number of laparoscopic procedures which would result in a decreased need for open-surgery instruments. However, changes in the surgical methods were not a part of the instrument optimization - therefore, we do not consider this to be the reason for the observed decrease in number of instruments. Additionally, we do not have data on the ratio of laparoscopic instruments to elaborate on this.

The question though is whether the reduction in number of instruments in each tray has resulted in an increased use of single-use instruments or an increased use of individually packaged reusable instruments. Unfortunately, we do not have data to substantiate these possible trends.

We observed a minor, non-significant variation in the total number of trays post-optimization, with changes ranging from a decrease of 18 % to an increase of 28 %. This suggests that the optimization needs for tray numbers and their structure vary across individual specialties. Hence, the study observed a reduction in the total number of unique tray types across all surgical specialties except for KOP and BUO. However, the comparison of tray types before and after optimization revealed a 11–15 % reduction in total number of instruments, indicating alterations in tray design. Additionally, some tray types were completely eliminated, such as in EOP, where 16 unique tray types were removed, resulting in a 100 % reduction in instruments in these tray types. Despite the increase in the total number of each unique tray type, the total number of instruments decreased. Thus, we contend that comparing the reduction in the total number of instruments is a more valid measure than assessing the reduction in instruments per tray due to the changes implemented in tray design, including consolidation, separation, modularization, and standardization.

The average reduction in total number of instruments in this study was 16 %, with variations ranging from 6 % in NKX to 25 % in YOP. However, to our knowledge, there is limited research reporting the total number of instruments before and after surgical instrument optimization. Moreover, in any optimization process it is imperative to consider the quantity and quality of instruments in the trays before optimization, as older and worn-out instruments are more likely to be replaced than newer, fully functional ones. This could explain some of the discrepancy in optimization studies in general, including those in the current study and the referenced ones. However, we found no overall pattern indicating that the condition of the instruments before optimization had any impact on the extent of the optimization process.

In this study, the decrease in the number of instruments reduced complexity and increased standardization. These aspects are expected to have a positive impact on OR preparation, setup time, time used handling instruments during surgery, and precleaning time in the OR room. Previous research supports these findings, demonstrating that a reduced number of instruments can lead to decreased setup and post-operative handling times. For instance, Farrokhi et al. [15] reported a significant decrease in setup time for minimally invasive spine surgery by 37 %. Similarly, Crosby et al. [6] found statistically significant reductions in setup time (26 % - 37 %) and tray assembly times (58 % - 66 %).

Furthermore, reducing the number of available instruments during surgery has been shown to mitigate the risk of errors in handing inaccurate or wrong instruments to surgeons [16]. Indicating that patient safety and efficiency in the OR will be positively affected by this instrument optimization.

The question of whether instrument optimization directly improve overall effectiveness in the OR remains unresolved. Based on previous studies reductions in setup times are expected to range from 5 to 15 min both before and after surgery [6,15]. Even though a day with multiple surgeries may potentially yield up to one hour of time savings, it may not be sufficient to accommodate an additional surgery into the schedule as most surgeries typically last over one hour.

However, factors such as scheduling and planning quality, individual surgeon efficiency [17], team cooperation and communication [16,18], and the patients' general health condition [17] are expected to exert a greater influence on OR effectiveness than solely reduced instrument setup time.

We anticipate that optimizing instrument sets with a reduction in the total number of instruments will result in resource savings, including manpower for cleaning, water, detergents, energy for washing, disinfection, and sterilization, as well as materials for manufacturing new instruments. However, our data do not include observations to investigate these issues further.

Therefore, the potential impact of reducing setup time by 5 to 15 min per surgery on workload in the CSSD at Aarhus University Hospital has yet to be confirmed. Further research and ongoing evaluation of OR processes are needed to fully assess the broader implications of instrument optimization on overall OR efficiency and resource utilization.

A strength of the current study is that it addresses set optimization across all specialties of a hospital, rather than focusing solely on individual specialties. Additionally, this study distinguishes itself from previous research by subjecting set optimization to thorough statistical analyses.

## Conclusion

This study underscores the complexity involved in instrument tray design, highlighting the absence of research demonstrating how to ensure reduced instrument usage across all surgical specialties in a large university hospital. The approach adopted at Aarhus University Hospital, which integrated various methods, demonstrated how interdisciplinary teams of experts, through an iterative design process, successfully achieved significant reductions in number of instruments. Our study emphasizes that comparing the reduction in the total number of instruments provides a more accurate assessment than comparing reductions in instruments per tray. Additionally, the data suggests that the reduction in instruments per tray and across multiple surgical disciplines can potentially translate into a decreased workload in the CSSD, offering an opportunity to reduce resources wasted on cleaning unused instruments in the CSSD.

## Funding sources

This research did not receive any specific grant from funding agencies in the public, commercial, or not-for-profit sectors.

## CRediT authorship contribution statement

**Peter Rubak:** Writing – review & editing, Writing – original draft, Visualization, Validation, Supervision, Project administration, Methodology, Investigation, Formal analysis, Conceptualization. **Ann-Eva Christensen:** Writing – review & editing, Writing – original draft, Visualization, Validation, Software, Methodology, Investigation, Formal analysis. **Mads Granlie:** Writing – review & editing, Writing – original draft, Validation, Methodology, Investigation, Formal analysis, Data curation, Conceptualization. **Karin Bundgaard:** Writing – review & editing, Writing – original draft, Visualization, Validation, Supervision, Resources, Project administration, Methodology, Investigation, Formal analysis, Conceptualization.

## Ethical approval

Approval to utilize the data from the optimization process was granted by the management of the Central Sterile Supply Specialty (CSSD) at Aarhus University Hospital. Neither ethics committee approval or informed consent were needed as no patients or volunteers (including organ/tissue donors) were involved in this study.

## Declaration of generative AI and AI-assisted technologies in the writing process

During the preparation of this work, all authors, who are non-native English speakers, utilized ChatGPT to enhance readability and language quality. After using this tool, the authors thoroughly reviewed and edited the content as needed and take full responsibility for the content of the publication.

## Declaration of competing interest

All the authors in this article have nothing to declare.
